# Quantitative Analysis of the Specific Absorption Rate Dependence on the Magnetic Field Strength in Zn_x_Fe_3−x_O_4_ Nanoparticles

**DOI:** 10.3390/ijms21207775

**Published:** 2020-10-21

**Authors:** Mohamed Alae Ait Kerroum, Cristian Iacovita, Walid Baaziz, Dris Ihiawakrim, Guillaume Rogez, Mohammed Benaissa, Constantin Mihai Lucaciu, Ovidiu Ersen

**Affiliations:** 1 Institut de Physique et Chimie des Matériaux de Strasbourg (IPCMS), UMR 7504 CNRS-Université de Strasbourg, 23 rue du Loess BP 43, 67034 Strasbourg CEDEX 2, France; mohamed-alae.ait-kerroum@ipcms.unistra.fr (M.A.A.K.); walid.baaziz@ipcms.unistra.fr (W.B.); dris.ihiawakrim@ipcms.unistra.fr (D.I.); guillaume.rogez@ipcms.unistra.fr (G.R.); 2 Laboratoire de Matière Condensée et Sciences Interdisciplinaires (LaMCScI), Faculty of Sciences, BP 1014 RP, Mohammed V University in Rabat, 10000 Rabat, Morocco; benaissa@fsr.ac.ma; 3 Department of Pharmaceutical Physics-Biophysics, Faculty of Pharmacy, “Iuliu Hatieganu” University of Medicine and Pharmacy, Pasteur 6, 400349 Cluj-Napoca, Romania; cristian.iacovita@umfcluj.ro

**Keywords:** zinc doped iron oxide magnetic nanoparticles, co-precipitation method, magnetic hyperthermia, specific absorption rate, Linear Response Theory, saturation of SAR, Neel relaxation time, Brown relaxation time

## Abstract

Superparamagnetic Zn_x_Fe_3−x_O_4_ magnetic nanoparticles (0 ≤ x < 0.5) with spherical shapes of 16 nm average diameter and different zinc doping level have been successfully synthesized by co-precipitation method. The homogeneous zinc substitution of iron cations into the magnetite crystalline structure has led to an increase in the saturation magnetization of nanoparticles up to 120 Am^2^/kg for x ~ 0.3. The specific absorption rate (SAR) values increased considerably when x is varied between 0 and 0.3 and then decreased for x ~ 0.5. The SAR values are reduced upon the immobilization of the nanoparticles in a solid matrix being significantly increased by a pre-alignment step in a uniform static magnetic field before immobilization. The SAR values displayed a quadratic dependence on the alternating magnetic field amplitude (H) up to 35 kA/m. Above this value, a clear saturation effect of SAR was observed that was successfully described qualitatively and quantitatively by considering the non-linear field’s effects and the magnetic field dependence of both Brown and Neel relaxation times. The Neel relaxation time depends more steeply on H as compared with the Brown relaxation time, and the magnetization relaxation might be dominated by the Neel mechanism, even for nanoparticles with large diameter.

## 1. Introduction

Advances in nanotechnology, especially in the synthesis methods, have enabled the development of magnetic nanoparticles (MNPs) with variable sizes—from few nanometres to several hundred nanometres—and different shapes, chemical composition, and surface functionalities [1,2,3,4,5,6]. The intrinsic magnetic properties of MNPs—significantly different from their bulk counterparts—in association with their high surface area to volume ratio have promoted them to be used in many applications, ranging from technology [7], to environmental science [8] and more recently to biotechnology and biomedicine [9,10,11,12,13]. In particular, the reduction of the MNPs size below a critical value drives the MNPs in the single-domain regime displaying a large constant magnetic moment and behaving like a giant paramagnetic atom, when the temperature is above the so-called blocking temperature (T_B_), which usually is situated far below the room temperature [14]. In the superparamagnetic (SP) regime, the MNPs respond fast to an external applied magnetic field and display negligible remanence (residual magnetism) and coercivity (the field required to bring the magnetization to zero). These intrinsic magnetic properties of the SP-MNPs annihilate the risk of forming agglomerates at room temperature making them very attractive for a broad range of biotechnological/biomedical applications [14,15].

Based on their additional properties, as biocompatibility and low toxicity profile and superior biodegradability [16,17,18], the US Food and Drug Administration has made from the SP iron oxide nanoparticles (often called SPIONs)—magnetite (Fe_3_O_4_) and maghemite (Fe_2_O_3_)—the only class of MNPs approved for clinical use [19] Several formulation based on SPIONs are already commercially available on the market, either as contrast agents in magnetic resonance imaging (MRI) applications (Lumiren^®^ for bowel imaging, Feridex IV^®^ and Resovist^®^ for liver and spleen imaging, Combidex^®^ for lymph node metastases imaging, and Ferumoxytol^®^ for iron replacement therapy [9]) or as heating agents in magnetic hyperthermia (MH) applications (Nanotherm^®^) [20].

One of the specific features of either SPIONs or MNPs, in general, is the high surface to volume ratio. This implies that a high number of atoms at the SPIONs surface have different coordination as well as dangling bonds, or undergo oxidation, giving rise to a surface shell of irregularly canted spins. This magnetically dead layer [21] leads to a drop in the saturation magnetization (M_s_) of the SPIONs [22] well below to their bulk counterparts (90 Am^2^/kg_metal_) [23]. Consequently, the SPIONs display reduced heating capabilities in MH and low relaxivity in MRI applications [24,25]. In these conditions, the implementation of SPIONs in MH applications has necessitated the simultaneous use of conventional therapies, as radiotherapy [26] or chemotherapy [27]. Alternatively, large quantities of SPIONs can be injected in tumours, which in turn might be associated with a high toxicity profile. Therefore, a huge interest was devoted to synthesizing MNPs with improved magnetic performances by keeping them in the SP limit, at room temperature [28,29].

Magnetite possesses an inverse cubic spinel structure where the divalent oxygen anions (O^2−^) form a face-centred cubic (FCC) lattice in which coexist two kinds of sublattices, namely: tetrahedral (A-sites)—occupied by trivalent cations Fe^3+^—and octahedral (B-sites)—shared by both divalent Fe^2+^ and trivalent Fe^3+^ cations. The divalent O^2−^ anions intermediate their different superexchange interactions between iron cations such as A-O-A, B-O-B, and A-O-B. The first two magnetic interactions align ferromagnetically the cations in each sublattice, whereas the last one is responsible for the ferrimagnetic feature manifested by the magnetite, which implies an antiferromagnetic coupling between the cations situated in both sublattices. As a consequence, the net magnetic moment is given by the difference between the moment in B and A sites (μ_oct_ − μ_tet_), namely, it comes from the divalent Fe^2+^ ions, which is 4μ_B_ (Bohr magneton) per formula unit. Therefore, an efficient way to modulate the magnetism of Fe_3_O_4_ can be achieved by replacing in its inverse cubic spinel structure the Fe^2+^ ions with common divalent transition metal cations as Mn^2+^, Fe^2+^, Co^2+^, Ni^2+^, Cu^2+^, and Zn^2+^. It has been shown that the substitution of Fe^2+^ (d^6^) cations in B sites with Mn^2+^ (d^5^) cations, bearing a larger magnetic moment compared with Fe^2+^, led to an increase of the overall magnetic moment from 4μ_B_ to 5μ_B_ per formula unit, providing a higher mass magnetization (110 Am^2^/kg_metal_), and thus with increased heating and relaxivity capabilities [30].

A change in the M_s_ of SPIONs has been also achieved through zinc doping, although the divalent Zn^2+^ cations bear a zero magnetic moment [29]. The M_s_ of SPIONs depends drastically on the amount of zinc incorporated in the spinel structure of Fe_3_O_4_ MNPs [29]. If the Fe_3_O_4_ structure is doped with a low amount of Zn^2+^ cations, they preferentially occupy A sites, thus, forcing the trivalent Fe^3+^ cations to migrate to B sites. Consequently, the μ_oct_ increases while the μ_tet_ decreases, resulting in a substantial increment of the M_s_ of 15 nm spherical Zn_x_Fe_3−x_O_4_ MNPs (161 Am^2^/kg_metal_ for x ~ 0.4) compared to both Fe_3_O_4_ (90 Am^2^/kg_metal_) and MnFe_2_O_4_ (110 Am^2^/kg_metal_) MNPs of the same size [31]. This type of MNPs exhibit 4 times greater heating capabilities and 10-fold higher relaxivity than that of conventional SPIONs. As the doping level of Zn^2+^ ions is increased (x > 0.5) the antiferromagnetic coupling between trivalent Fe^3+^ cations in B sites is more favourable and consequently, the M_s_ of Zn_x_Fe_3−x_O_4_ MNPs drops to lower values [31].

The most salient disadvantage of the above mentioned highly performant Zn_x_Fe_3−x_O_4_ MNPs is that they have been obtained in nonpolar solvents using thermal decomposition techniques, which requires subsequent post-synthesis treatments, to make the MNPs hydrophilic and render them biocompatible. These treatments might perturb their heating or relaxivity performances [32]. Therefore, a large number of other synthesis techniques, capable to generate water-dispersible MNPs in a single step, have been reported in the literature to elaborate Zn doped magnetite. For instance, the biogenic approach has led to the formation of Zn_0.16_Fe_2.84_O_4_ MNPs with an M_s_ of 100 Am^2^/kg_metal_ at room temperature exhibiting a relaxivity 5.2 times higher than the Feridex IV^®^ [33]. Hydrothermal reduction route, solvothermal method, and microwave refluxing technique have also demonstrated the formation of non-stoichiometric Zn_x_Fe_3−x_O_4_ MNPs with enhanced M_s_ as zinc contents increases in the range 0 < x < 0.4 [34,35,36]. The most popular synthesis route employed for the synthesis of zinc doped magnetite was by far the co-precipitation method due to its simplicity, fastness, scalability and high quality of the resulting MNPs in terms of purity and crystallinity [37]. Different issues have been addressed as the effective elaboration of Zn_x_Fe_3−x_O_4_ MNPs employing this technique [38,39], the effect of crystal size on the magnetic behaviour of Zn_x_Fe_3−x_O_4_ MNPs [40], the effect of the base (NaOH and NH_3_) on the zinc incorporation into the crystalline structure [41], and recently the synergic effect of both size and zinc doping level on the magnetic properties of Zn_x_Fe_3−x_O_4_ MNPs [42].

In the present work, we propose to relate the magnetic and heating properties of Zn doped magnetite NPs to the Zn content. In this sense, we synthesized a series of magnetite and zinc doped magnetite NPs through co-precipitation, with different amounts of zinc keeping unchanged their size, crystallinity, polydispersity, and morphology. This kind of approach helps us to better understand how the amount of zinc doping is reflected in the MNPs structural, magnetic and hyperthermic properties and, in this manner, to discriminate the parameters which are significantly influencing these properties. By carefully adjusting the amount of magnetic precursors and the pH in the synthesis method, citric acid capped spherical Fe_3_O_4_ and Zn_x_Fe_3−x_O_4_ MNPs, with different zinc amount (0 < x < 0.5), displaying an average diameter around 16 nm, similar size distribution and crystalline size have been elaborated. The MNPs were completely characterized by several physicochemical techniques. Finally, their magnetic and hyperthermic properties were assessed. Taking advantage of our hyperthermia set-up, with high alternating magnetic field (AMF) strength amplitudes, we were able to demonstrate that the heat released by the MNPs saturates at AMF amplitudes above 35 kA/m. This type of behaviour is unusual for SPIONs for which it is assumed that their heating capabilities increase with the square of the AMF strength amplitude. Developing a model in which, at high magnetic field amplitudes, the magnetization follows a Langevin type dependence on the field strength and, considering the field dependence of both the Neel and Brown relaxation times, we were able to describe this saturation.

The specific absorption rate (SAR) values of the MNPs, the parameter that characterizes their heating capabilities, is the result of several heating mechanisms. In the case of SPIONs, two relaxation phenomena are considered responsible for the heat released: the Neel relaxation mechanism by which the magnetic momentum orientation of the MNPs follows the changes in the external AMF, and the Brown relaxation, by which the MNPs physically rotates following the change in the external AMF. We performed hyperthermia measurements both in water for a colloidal suspension of the MNPs and as a solid dispersion in polyethylene glycol 8000 (PEG 8k) (frozen bellow 60 °C) aiming at evaluating the contribution of Brown relaxation mechanism to the MNPs overall heating efficiency. We also took advantage of this particular setup, in which the MNPs were initially uniformly dispersed in liquid PEG 8k at 80 °C, and after that, the suspensions were frozen and cooled down to room temperature to study the effect of the MNPs alignment in a DC magnetic field. In this sense, measurements were performed on both randomly dispersed MNPs or MNPs aligned in an external static magnetic field, before and during the freezing of the sample demonstrating that the pre-alignment of the samples in static magnetic fields significantly increases their SAR values.

## 2. Results and Discussion

### 2.1. Morphological Properties

**Figure 1:**
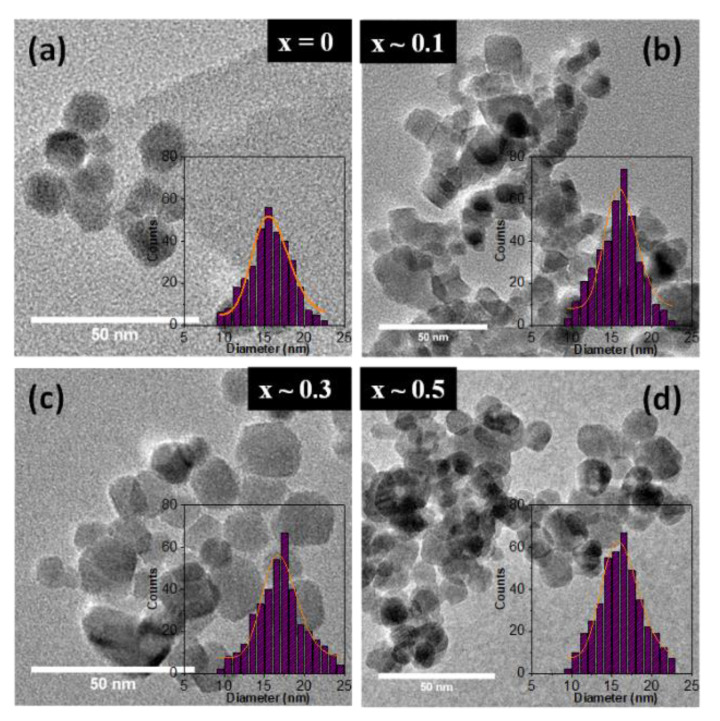
TEM images of Zn_x_Fe_3−x_O_4_ MNPs with different stoichiometry (**a**) x ~ 0, (**b**) x ~ 0.1 (**c**) x ~ 0.3 and (**d**) x ~ 0.5. Insets represent size distribution histograms of Zn_x_Fe_3−x_O_4_ MNPs fitted to a log-normal distribution (orange lines).

Typical TEM micrographs of the synthesized Zn_x_Fe_3−x_O_4_ MNPs with 0 ≤ x < 0.5 along with their corresponding size distribution histograms are shown in Figure 1. The mean size of MNPs was estimated by adjusting the experimental histograms (obtained from statistics of about 500 MNPs) with a log-normal function, and the results are shown in Table 1. As presented above, all MNPs are synthesized in the same conditions, which means at a temperature of 80 °C and a pH value of 12. In the absence of zinc doping, irregular, almost spherical iron oxide MNPs have been formed, with an average diameter of about 16 nm (Figure 1a). As the zinc content is increased in the synthesis from 0.44 mmol to 2.2 mmol, the resulting nonstoichiometric Zn_x_Fe_3−x_O_4_ MNPs (0 ≤ x < 0.5) keep an overall spherical shape with some MNPs exhibiting nonetheless a rectangular shape (Figure 1b–d). For these MNPs, the mean edge was considering to be the specific size, and as it can be seen in Table 1, a slight increase in the average size within 1 nm can be assigned to the zinc doped MNPs. The polydispersity index of all four types of MNPs varies between 0.12 and 0.14 indicating a quasi-narrow size distribution. Therefore, the precise control of the amounts of magnetic precursor in the co-precipitation method able to generate zinc doped Fe_3_O_4_ MNPs (with 0 ≤ x < 0.5) with an overall spherical shape of the same diameter (16–17 nm). This will allow us to study more accurately the effect of zinc doping level on the structural, magnetic and hyperthermia properties of the synthesized Zn_x_Fe_3−x_O_4_ MNPs.

### 2.2. Structural Properties

**Figure 2:**
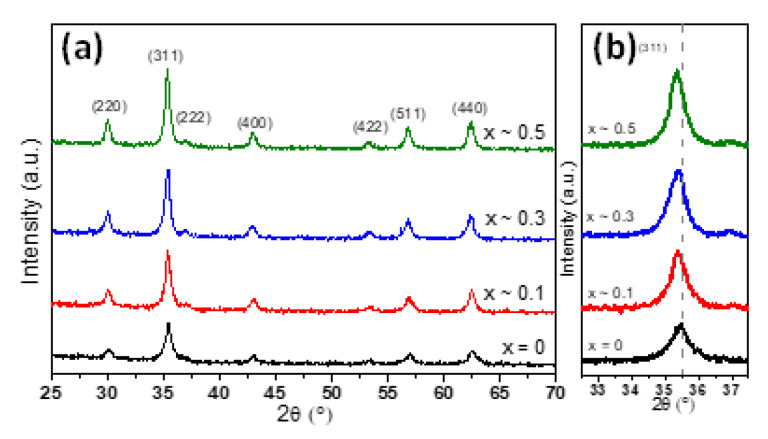
(**a**) XRD diffraction patterns and (**b**) zooms in the (311) diffraction peak region of Zn_x_Fe_3−x_O_4_ MNPs with different stoichiometry (0 ≤ x < 0.5).

The crystalline structure of MNPs has been evaluated by X-ray diffraction (XRD) performed on powder samples (Figure 2). In the case of undoped zinc MNPs, the XRD pattern (black diffractogram in Figure 2a) revealed the existence of a single-phase cubic spinel crystalline structure. The position and the relative intensities of all diffraction peaks can be assigned to (220), (311), (222), (400), (422), (511) and (440) crystallographic planes, which match the JCPDS card no. 01-080-6402 corresponding to the cubic spinel crystal structure of Fe_3_O_4_. No other peaks were detected within the limit of observation in the XRD pattern, confirming the high purity of the Fe_3_O_4_ MNPs. Moreover, the average lattice constant for the Fe_3_O_4_ MNPs was found to be a = 8.393 Å, which is closed to the bulk magnetite (a = 8.396 Å) and not to that of maghemite (a = 8.3315 Å). The absence of extra peaks at (210) and (211) in the XRD pattern assigned to maghemite and the black colour of the powder further confirmed that the Fe_3_O_4_ MNPs have been successfully synthesized. The XRD patterns corresponding to the zinc substituted MNPs confirmed the presence of magnetite in all diffractograms (Figure 2a). The peaks assigned to (220), (311), (222), (400), (422), (511) and (440) crystallographic planes are well defined being the only ones present in the diffractograms (Figure 2a). No observable diffraction peaks correspond to zinc oxide (ZnO), thus suggesting that the zinc would be part of the crystalline structure of magnetite. A closer look at all XRD patterns revealed that the diffraction peaks were progressively shifted towards lower angles with increased zinc substitution in Zn_x_Fe_3−x_O_4_ MNPs. This trend can be clearly seen in Figure 2b for the more intense diffraction peak (311) and in the Supplementary Figure S1 for other three main diffraction peaks which are (220), (511) and (440). Our results are in perfect agreement with the shifts generated by Zn_x_Fe_3−x_O_4_ MNPs of different sizes but similar compositions [32,33,36,40]. As demonstrated in those studies, the shifts of diffraction peaks of Zn_x_Fe_3−x_O_4_ MNPs with respect to those of Fe_3_O_4_ MNPs represent a clear evidence that the Zn^2+^ ions prefer to occupy the tetrahedral (A) sites, forcing the Fe^3+^ ions to migrate to octahedral (B) sites and replace the divalent Fe^2+^ ions.

According to Table 1, the corresponding lattice parameter was found increasing from a = 8.393 Å to a = 8.412 Å as the zinc concentration in magnetite increased in the range 0 < x < 0.5. The increase in lattice parameter a with increasing zinc concentration was also reported by other authors either for zinc doped magnetite MNPs [32,33,36,40,42] or for zinc doped cobalt ferrite MNPs [43]. This trend is ascribed to the larger radius of Zn^2+^ (0.74 Å) ions compared to trivalent Fe^3+^ (0.49 Å) or divalent Fe^2+^ ions (0.61 Å) [44]. Since the tetrahedral (A) sites are smaller than the octahedral (B) sites, the preferential occupancy of Zn^2+^ ions of A sites leads to an expansion of the crystalline structure, thus to an increase of the lattice parameter value. It can be observed that the spinel peaks for all four types of MNPs are broad, implying the formation of nano-sized crystals. The average crystalline size for Zn_x_Fe_3−x_O_4_ (0 ≤ x < 0.5) MNPs was calculated using Scherrer’s formula by Gaussian fit of the peaks (220), (311) and (440). The results, listed in Table 1, show that the D_XRD_ slightly increases by increasing the zinc content up to x ~ 0.3 and then it decreases by further increasing of zinc content up to x ~ 0.5. The same trend is observed for the average diameter (D_TEM_) obtained from TEM histograms. As usually, in the case of all four types of MNPs, the D_TEM_ values are slightly higher than the D_XRD_ (Table 1). Note that the size revealed by the XRD data corresponds to the smallest crystallites, as these crystallites are those which give the largest broadening in the XRD peaks. The relatively narrow size distribution histograms of all four types of MNPs (Figure 1) and the fact that the D_XRD_ values are very close to the D_TEM_ values suggest that most of the MNPs are single crystals.

**Figure 3:**
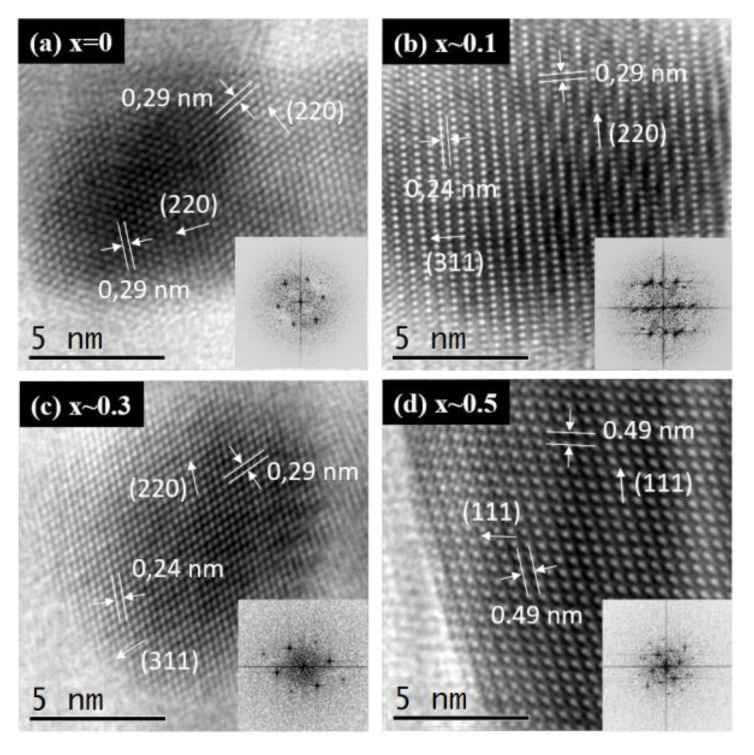
High resolution TEM images of Zn_x_Fe_3−x_O_4_ MNPs with different stoichiometry (**a**) x ~ 0, (**b**) x ~ 0.1, (**c**) x ~ 0.3 and (**d**) x ~ 0.5. The inset represents the 2D Fast Fourier Transform (FFT).

The microstructure features of the Zn_x_Fe_3−x_O_4_ MNPs with different zinc content were revealed by high-resolution TEM images acquired on single MNPs (Figure 3a–d). These images exhibit atomic resolution demonstrating the high crystallinity of the synthesized zinc substituted Fe_3_O_4_ MNPs. The orientation of different atomic planes among single MNPs is clearly visible making it very easy to identify the interatomic distance between them. The high resolution-TEM images underline the presence of several crystalline plans (d_hkl_) depending on the orientation of the single MNPs with respect to the direction of the electron beam. The following d_hkl_ have been measured and are 0.49, 0.29 and 0.24 nm, corresponding, respectively, to the (111), (220) and (311) plans of the spinel structure of magnetite (JCPDS card no. 01-080-6402) and zinc ferrite (JCPDS card no. 00-022-1012). As the (111) reflection displays a low relative intensity, the distance of 0.49 nm primarily assigned to the (111) plans can be also assigned to (311) plans, in combination with a Moiré effect. From these data, it is difficult to conclude on the insertion of Zn^2+^ ions inside the Fe_3_O_4_ structure, as the slightly higher apparent size of the Zn^2+^ ions and their small relative amount are probably not enough to influence considerably the crystallographic distances of the three type of Zn_x_Fe_3−x_O_4_ MNPs, for which the corresponding distances remain similar to those of stoichiometric magnetite and zinc ferrite MNPs. The frequency spots corresponding to crystallographic planes can be easily visualized on the Fast Fourier Transform micrographs (insets of Figure 3a–d) and are consistent with the different d_hkl_ characteristics of the spinel structure of zinc doped iron oxide ferrite.

**Figure 4:**
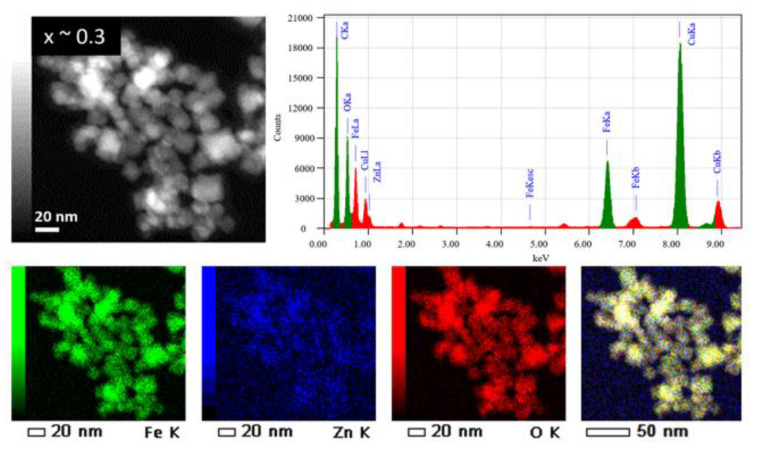
(Upper panels) TEM image of Zn_x_Fe_3−x_O_4_ MNPs with x ~ 0.3 and the corresponding EDX spectrum (C and Cu signals from the TEM grid are also observed). (Lower panels) from left to right: chemical maps of Fe, Zn, O and a global chemical map with the distribution of the 3 elements (Fe, Zn and O).

The chemical composition of the Zn_x_Fe_3−x_O_4_ MNPs was determined by using energy dispersive X-ray (EDX) analysis carried out on typical MNPs belonging to the 3 samples with different zinc contents (x ~ 0.1, ~ 0.3 and x ~ 0.5). The EDX spectra and EDX maps of Fe, Zn and O elements in the Zn_x_Fe_3−x_O_4_ MNPs from the sample x ~ 0.3 are shown in Figure 4, while EDX results of the samples with x ~ 0.1 and x ~ 0.5 are summarized in Supplementary Figure S2. It is worth noting that for all these samples, the Fe and Zn are found to be homogenously distributed within the total volume of the MNPs [31] and/or of the aggregate; no core-shell structures or MNPs containing only Fe or Zn were observed. Such results involve a quite simultaneous decomposition process of both iron and zinc magnetic precursors in the reaction medium.

The EDX analysis enables us to quantify both Fe and Zn atomic percentage within each type of sample. That means to estimate the “x” value and thus the real composition of the Zn_x_Fe_3−x_O_4_ at the single MNPs level. For the three Zn_x_Fe_3−x_O_4_ samples, the quantitative analysis of the EDX spectra provides the “x” values of 0.09, 0.27 and 0.45 instead of the theoretical values of 0.1, 0.3 and 0.5, respectively (Table 2). The zinc atomic percentage in each sample appears thus slightly lower than the calculated ones.

### 2.3. Biofunctionalisation of MNPs

**Figure 5:**
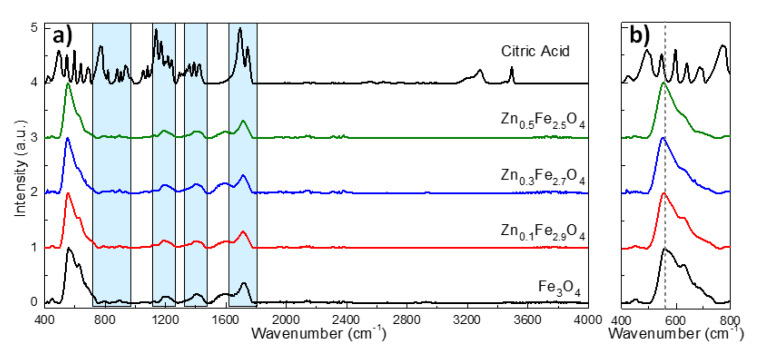
(**a**) FT-IR spectra of citric acid and citric acid coated Zn_x_Fe_3−x_O_4_ MNPs (0 ≤ x < 0.5) and (**b**) Zoom in the 400–800 cm^−1^ region of FT-IR spectra. The spectra are normalized to the highest absorption band and shifted for clarity.

The FT-IR spectra of the citric acid-coated MNPs along with pure citric acid powder are provided in Figure 5. Pure citric acid exhibits many characteristic absorptions bands in the wavenumber range between 400 and 1800 cm^−1^. The most relevant absorption bands for our studies are those located around 1700, 1400 and 1200 and 900 cm^−1^ assignable to the stretching vibration of C=O from the COOH group, symmetric stretching of COO^−^, symmetric stretching of C–O, and OH group of citric acid, respectively. As marked by the blue rectangles in Figure 5a, the mentioned groups of absorption bands are clearly visible in the FT-IR spectra of citric acid coated MNPs, exception made of those absorption bands between 700 and 1000 cm^−1^ which are faintly visible. Moreover, all these absorptions bands are shifted to lower wavenumbers and broaden on the FT-IR spectra of citric acid coated MNPs with respect to the FT-IR spectrum of the citric acid (black line in Figure 5a) [34,45,46]. Moreover, these absorption bands are absent in the FT-IR spectra of uncoated MNPs (Supplementary Figure S3a). This is a clear indication that the citric acid forms a complex with the Fe atoms on the MNPs surface through its carboxylate groups. The FT-IR spectra of either citric acid coated MNPs (Figure 5a) or uncoated MNPs (Supplementary Figure S3a) are dominated by absorption bands around 560 and 630 cm^−1^ which are attributed to the stretching vibration mode associated with the Zn^2+^-O and Fe^3+^-O in A site. These two pronounced absorption bands confirm the formation of the spinel structure, characterizing, in particular, the ferrites [32,42]. As indicated by the dotted line in Figure 5b and Supplementary Figure S3b, the absorption band that appears at 560 cm^−1^ in the FT-IR spectrum of Fe_3_O_4_ shifts progressively towards lower wavenumbers at 557, 554 and 552 cm^−1^ as the zinc doping is increased from 0.1 to 0.3 and 0.5, respectively. The decrease of the frequency of the absorption band related to the A site means an increase of the mass of cations located in A sites. This is possible only by replacing the iron atoms in A sites (55.85 amu) with heavier zinc atoms (65.38 amu) [42].

In addition, the citric acid coating improved considerably the colloidal stability of MNPs. Uncoated MNPs sedimented at the bottom of vials within several hours, while coated MNPs preserve their colloidal stability for a long time (10 days). At this point in time, the formation of a colour gradient is observed within the colloidal solution, which suggests size-dependent sedimentation of MNPs due to the gravity effect. In relation with this direct observation, dynamic light scattering (DLS) measurements show that the size distributions of all four samples exhibit two peaks, more exactly a well-defined peak and a shoulder (Supplementary Figure S4a). The prominent peak indicates that a large number of MNPs within the colloidal solution present a hydrodynamic diameter between 28 and 43 nm which correspond most probable to single MNPs. The second peak, which is less intense (being present as a shoulder of the first peak for two samples), indeed indicates the presence of aggregates of MNPs in the solution. Individual MNPs and their association in clusters are visible also in the typical TEM image (see Supplementary Figure S4b) recorded after coating, as well.

### 2.4. Magnetic Properties

**Figure 6:**
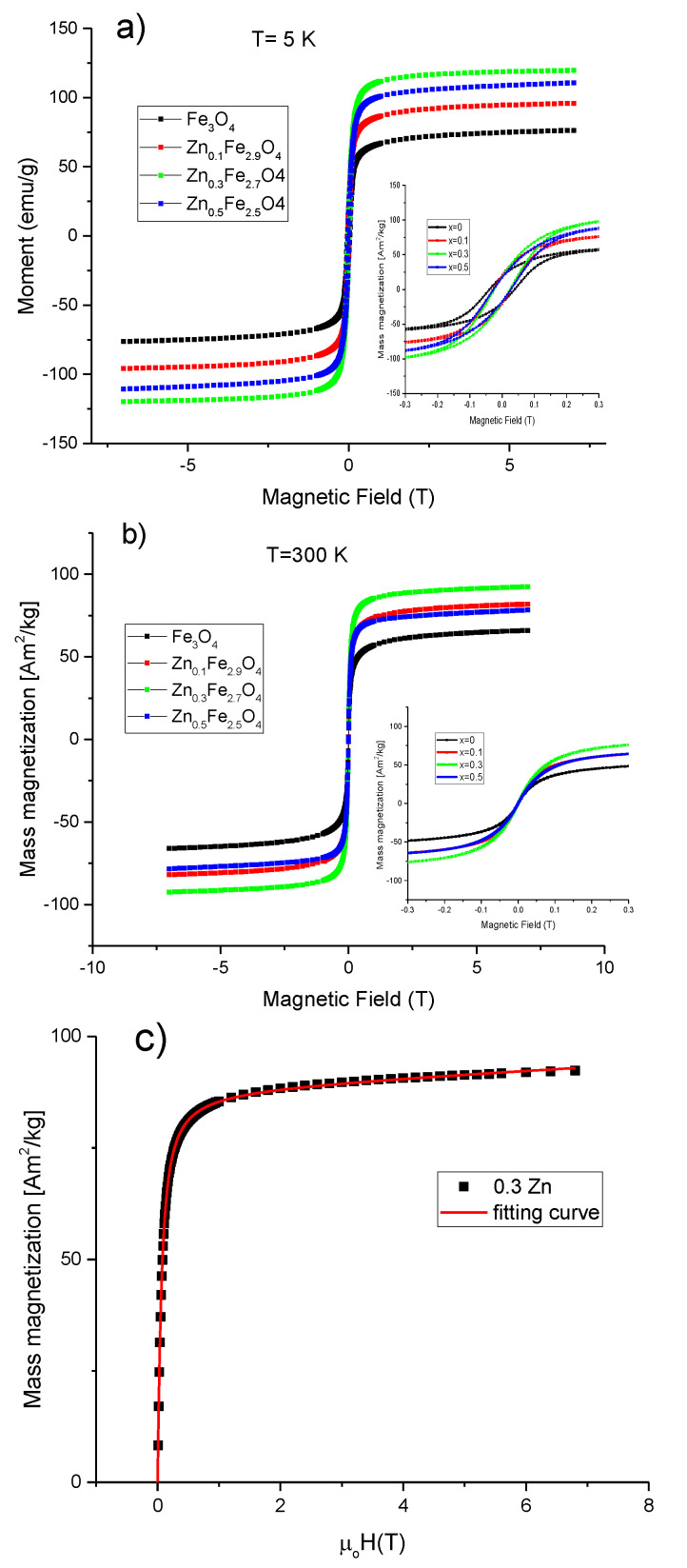
Magnetization curves for Zn_x_Fe_3−x_O_4_ MNPs (0 ≤ x < 0.5) at (**a**) 5 K and (**b**) 300 K. Insets represents low-field regime hysteresis loops. (**c**) Magnetization curve fitting for the Zn_0.3_Fe_2.7_O_4_ MNPs at 300 K; the blue squares represent the experimental data and the orange line is the fitting curve.

The evolution of the magnetic properties of MNPs as a function of the zinc doping level was monitored by SQUID magnetometry. In this regard, hysteresis loops have been collected at both low temperature (5 K) and room temperature (300 K) between ±70 T. The maximum magnetization reachable (M_s_) by the MNPs in the presence of an applied magnetic field, the coercive field (H_c_), the magnetic remanence (M_r_) together with the calculated anisotropy constants are collected in Table 3. At low temperature (5 K), all four types of MNPs showed hysteresis curves with similar shapes (Figure 6a), revealing the standard ferrimagnetic character. The Fe_3_O_4_ MNPs displayed M_s_ of 76.4 Am^2^/kg, which is slightly higher than other magnetite MNPs synthesized by co-precipitation [47] and significantly lower than the value usually reported for bulk material (92 Am^2^/kg). This small value of M_s_ compared to the bulk state might be attributed to the cation vacancies resulted from synthesis, but also to the surface effects as the oxidation and the presence of spin canted layer at the MNPs surface [48]. The incorporation of zinc into the magnetite structure led initially to an increase in M_s_ before decreasing again. For a small amount of zinc (x ~ 0.1), the M_s_ increased by 21% to 97.1 Am^2^/kg reaching a maximum of 120.1 Am^2^/kg for x ~ 0.3 (36% increase). For x ~ 0.5, the value of M_s_ decreases slightly to 110.1 Am^2^/kg, this value being still higher than that of magnetite MNPs (x = 0) by 31%. It is worth noting that these values of M_s_ are by far the highest reported for zinc doped magnetite MNPs synthesized by the co-precipitation method [42]. Taking into account that all four types of MNPs display similar morphological and structural properties, the significant enhancement in the M_s_ for zinc doped magnetite MNPs as compared to magnetite MNPs can only be attributed to the location of the divalent Zn^2+^ ions at the A sites of the spinel structure. The enhancement of the mass magnetization of the Zn_x_Fe_3−x_O_4_ (0 < x < 0.5) can be explained in the framework of Neel’s model of ferrimagnetism [49], where the dilution of the magnetic moment at the A sublattice (by the replacement of trivalent Fe^3+^ ions by non-magnetic Zn^2+^ ions), and the increase of the magnetic moment at the B sublattice (by the substitution of the divalent Fe^2+^ ions with 4 unpaired d-electrons with the trivalent Fe^3+^ ions with 5 unpaired d-electrons form the A sublattice) resulted in an important increment of the magnetic moment per formula unit (μ = μ_oct_ – μ_tet_). At a zinc doping level around 0.5, the μ_tet_ became so much diluted that the strength of antiferromagnetic A-O-B superexchange interactions started to weaken. Consequently, as explained by the Yafet and Kittel three sublattices model [50], the spins in B sublattice undergo antiferromagnetic coupling through B-O-B superexchange interaction leading to a reduction of the magnetic moment per formula unit (μ = μ_oct_ – μ_tet_) and thus M_s_ diminished.

The H_c_ obtained for magnetite MNPs at 4 K was 31 kA/m. As shown in Table 3, the H_c_ decreased to 23 kA/mfor x ~ 0.1, reaching a minimum of 19.1 kA/m for x ~ 0.3 and increased again to 21.5 kA/m by further increasing the zinc doping level to x ~ 0.5. Basically, the zinc doping of magnetite led to a slight decrease in the H_c_ of MNPs. The reduction of H_c_ for zinc-containing magnetite MNPs with respect to the unsubstituted magnetite MNPs was also reported for biogenic zinc doped magnetite MNPs [33] and zinc substituted cobalt ferrite MNPs [43]. The reduced values of H_c_ suggest a decrease of the magnetocrystalline anisotropy of zinc doped magnetite MNPs. As shown in Table 3, the ratios M_r_/M_s_ were smaller than 0.5 for all four types of MNPs indicating a uniaxial anisotropy. Therefore, the effective anisotropy constants were calculated according to the formula: K_eff_ = μ_0_H_c_M_s_/0.96 [50], where the M_s_ and the H_c_ values measured at 5K were employed. It was obtained a K_eff_ value of 1.6 × 10^4^ J/m^3^ for the spherical magnetite MNPs with a mean diameter around 16 nm, revealing their excellent crystalline quality. Slightly smaller values of the K_eff_ were obtained for the zinc substitute magnetite MNPs of the same size and shape. These values of K_eff_ are not consistent with those recently reported on Zn_x_Fe_3−x_O_4_ MNPs (0 ≤ x ≤ 0.5) synthesized by the co-precipitation method [42]. It has to be noted that as the zinc doping level is increased up to x ~ 0.3, the crystalline size of MNPs increases as well, leading to an increase of the K_eff_ [42]. In other words, the variation of K_eff_ was mainly attributed to the variation of the crystalline size of MNPs, while the zinc doping level has no effect. However, in our case, the zinc doping of magnetite MNPs, displaying similar shape and constant crystalline size, slightly modifies the H_c_ and K_eff_ of the MNPs. Nevertheless, the only magnetic parameter that significantly varies is the M_s_, as a consequence of the incorporation of the divalent Zn^2+^ ions at the A sites of the spinel structure of magnetite MNPs.

The room temperatures hysteresis loops for all four types of MNPs are presented in Figure 6b. As can be seen in Table 3, a reduction of M_s_ at room temperature was recorded, a behaviour that is typical for MNPs. In the case of magnetite MNPs, the difference between the M_s_ values at 5 K and 300 K was only 10.3 Am^2^/kg indicating the good crystallinity of MNPs. The difference in M_s_ increased progressively from 17.6 Am^2^/kg to 28 Am^2^/kg as zinc content in magnetite MNPs increased from x ~ 0.1 to x ~ 0.5, suggesting that the spin-canting effects are more pronounced for a higher content of zinc in magnetite MNPs. The trend of M_s_ as a function of the zinc doping level at 300 K was similar to that at 5 K. As compared to magnetite MNPs, the M_s_ increased by 20% to 78.5 Am^2^/kg for a small amount of zinc (x ~ 0.1), reached a maximum of 93.4 Am^2^/kg for x ~ 0.3 (41% increase) and decreased to 82.1 Am^2^/kg for x ~ 0.5 (24% increase). Non-zero coercivities (0.7–1 kA/m) were observed from the room temperatures hysteresis loops for all four types of MNPs. On the other hand, as can be seen that from Supplementary Figure S5, the zero-field-cooled/field-cooled (ZFC/FC) magnetization curves start to join slightly below 300 K, while the maximum in ZFC curves, which corresponds to the average onset of the ferromagnetic to superparamagnetic transition, is located at the same temperature. The maximum in ZFC is broadened, suggesting that the gradual transition to the SP state is extended over 300 K; hence, most of the MNPs can be defined as to be SP at room temperature.

The mean magnetic diameter of the MNPs, based on their magnetization saturation curves measured at 300 K, can be calculated assuming a log-normal distribution and using the following integral fitting equation:

(1) M=Ms∫0∞12π σDexp[−ln(DD0)22σ2]L(ξ)dD+χH

where *L*(*ξ*) = *coth ξ* − 1/*ξ*, is the Langevin function with its dimensionless argument *ξ* *=* *μ*_0_*M_s_V_m_H/k_B_T* with *μ*_0_ the vacuum magnetic permeability, *M_s_* the saturation magnetization, *H* magnetic field intensity, *V_m_ = πD*^3^*/6* the magnetic volume of the MNP, *D* the MNP diameter, and *σ* is the MNPs dispersivity index. The paramagnetic term in Equation (1) accounts for paramagnetic impurities and the very fine SP MNPs and improves significantly the fitting parameters with R^2^ > 0.999. The mean diameters obtained with this fitting procedure (*D_mean_ = D*_0_ exp(*σ*^2^/2)) are included in Table 1. A typical fitting curve is presented in Figure 6c for the sample Zn_0.3_Fe_2.7_O_4_ and in Supplementary Figure S6 for the other three samples. As expected, the mean magnetic diameters (around 8–9 nm) are significantly smaller as compared to those derived from TEM and XRD data, a phenomenon which is explained by the existence of the magnetic dead layers (thickness around 3 nm) and the spin canting effects [48].

### 2.5. Hyperthermia Properties

The heating of small superparamagnetic MNPs in small amplitude AMF (such as the Zeeman energy, which is smaller than the thermal energy) is described in the frame of the Linear Response Theory (LRT). Within this model proposed by Rosensweig [51], it is supposed that the magnetization of the nanoparticles depends linearly on the applied magnetic field, the proportionality factor being the complex susceptibility. The rate of volumetric heat release in an AMF can be written as

(2) $$P=\mu_{0}\pi \chi^{\prime \prime }fH_{max}^{2}$$

where *μ*_0_ is the vacuum magnetic permeability, *f* the frequency, *H_max_* the amplitude of the AMF strength, and *χ*″ the imaginary part of the magnetic susceptibility:

(3) $$\chi^{\prime \prime }=\chi_{0}\frac{\omega \tau }{1+{\left(\omega \tau \right)}^{2}}$$

with the static susceptibility,

(4) $$\chi_{0}=\frac{\mu_{0}M_{s}^{2}V}{3k_{B}T}$$

where *M_s_* is the saturation magnetization of the material, *V* its magnetic volume, *k_B_* is the Boltzmann constant and *T* absolute temperature. *τ* is the relaxation time and in the case when both Brown and Neel relaxations occur,

(5) $$\frac{1}{\tau }=\frac{1}{\tau_{N}}+\frac{1}{\tau_{B}}\;\;$$

where *τ_N_* and *τ_B_* are the Neel and Brown relaxation times. The overall relaxation time will always be smaller than any of the two relaxation times, the smaller relaxation time dictating its value. For small MNPs, with diameters below a critical value (depending however on the frequency of the alternating field and the viscosity of the medium), the Neel relaxation prevails [51] while for MNPs with larger diameters the Brown mechanism is dominant. The main result of the LRT is that the maximum power release is obtained when the angular frequency equals the reverse of the relaxation time *ωτ*  =  1, and the imaginary part of the susceptibility presents a maximum. Another feature predicted by the LRT is the square dependence of the SAR on the amplitude of the AMF strength without any saturation effect. The LRT model is limited to small applied field strengths for which the magnetic energy is smaller than the thermal energy *μH < k_B_T*. For higher magnetic field amplitudes, other approaches, like field-dependent relaxation times, need to be used to explain the saturation (as will be discussed later).

**Figure 7:**
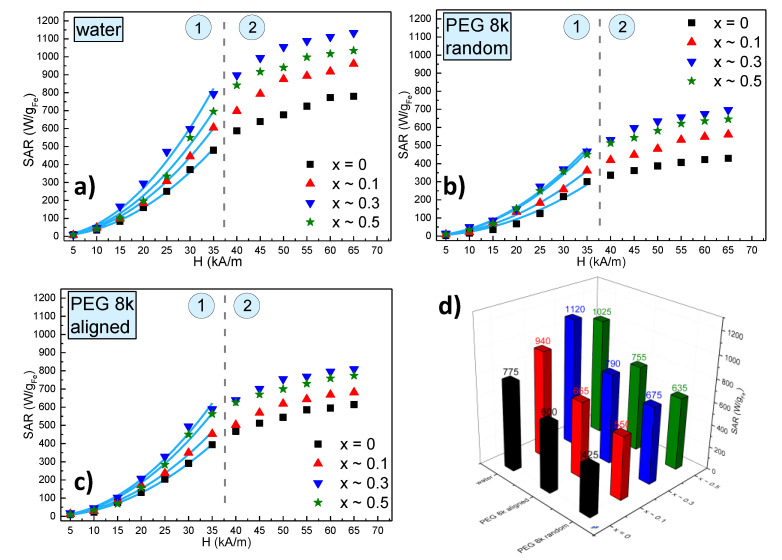
Specific absorption rate (SAR) dependence on the AMF amplitude (H) for Zn_x_Fe_3−x_O_4_ MNPs (0 ≤ x < 0.5) (**a**) in water, (**b**) in solid polyethylene glycol 8000 (PEG 8k) with a random orientation and (**c**) in a solid PEG 8k pre-aligned parallel with alternating magnetic field directions. (**d**) The saturation values of SAR for all 4 samples in water and PEG 8k both in random and aligned organizations. The dashed green lines delimit the two regions of the SAR evolution with H, which are marked by the numbers 1 and 2. The continuous blue lines represent fittings with the parabolic dependence of SAR on H.

The SAR dependence on the amplitude of the AMF strength for all 4 types of MNPs in water is presented in Figure 7a, while the corresponding heating curves are shown in Supplementary Figure S7. One may notice that as the AMF amplitude increases the SAR values increase but there are at least two types of behaviours: at low AMF amplitudes (0–35 kA/m) SAR increases with the square of the AMF amplitude; above 35 kA/m (40–65 kA/m) a clear saturation effect was observed. In both regions, the SAR values are higher for zinc doped ferrites as compared to the pure magnetite sample. However, the SAR dependence on the doping level is non-monotonous, i.e., SAR increases as x is varied between 0 and 0.3 and then decreases for x ~ 0.5. A clear picture of this dependence can be seen in Figure 7d, where the saturation values of SAR are plotted as a function of the zinc doping level. In other words, these results are consistent with the evolution of the M_s_ as a function of the zinc doping values, M_s_ attains a maximum value for x ~ 0.3 and decreases again for x ~ 0.5. Please note that SAR corresponding to x ~ 0.5 is higher than the SAR at x ~ 0.1, similar to the values of M_s_ for the two samples. The perfect parallelism between the SAR and M_s_ behaviour against the zinc doping value is a clear indication that, at least for these samples, the SAR is mainly determined by the M_s_. This observation is also consistent with the fact that the samples at room temperature (300 K) have a negligible coercive field (the nonzero value probably being due to some MNPs with the largest sizes within a sample), and therefore, the only magnetic property which remains relevant at this temperature is the M_s_ together with the size. Because the sizes are similar for all 4 types of MNPs, their heating abilities are related to their M_s_. This can be seen from the linear dependence of all the SAR values on the M_s_ (Supplementary Figure S8).

One important issue in the in vivo application of the magnetic hyperthermia is that, in most of the cases, the synthesized MNPs had much better heating performances in vitro when they are uniformly dispersed, as compared to cell cultures or in vivo. In cell cultures, for example, it was observed that MNPs accumulate, agglomerate in endosomes and their heating characteristics drastically decline. This decrease in the heating performance was related both to an increase in the dipolar interaction of agglomerated/aggregated MNPs and the immobilization of the MNPs either in the endosomes or the cytosol of the cells, blocking the Brown relaxation mechanisms [52]. For these reasons, it was suggested that the in vivo heating ability of MNPs should be tested with immobilized particles, which means with blocked Brown relaxation mechanism as it does not contribute to the SAR values in these conditions [53]. In our previous works [54,55], we were able to discriminate different heating mechanisms of MNPs by dispersing them either in highly viscous PEG1k or in solid PEG8k (with a prior melting), demonstrating that this immobilization leads to a significant decrease of the SAR. In our case, the four types of MNPs synthesized in this work were uniformly dispersed in liquid PEG8k at 80 °C, and afterwards, the samples were allowed to cool down to room temperature (Supplementary Figure S9). As can be seen from Figure 7b,d, the SAR values decrease significantly for all four samples. The decrease of the SAR values is almost the same in all four samples (about 30%) as can be seen from Supplementary Figure S8 were the lines representing the linear dependence of SAR on M_s_ are almost parallel for the samples measured in water and solid PEG 8k.

This specific set-up of uniformly dispersed MNPs in a liquid before being frozen, allowed us to measure the effect of aligning the MNP before their immobilization within the sample. The interest in studying the effects of pre-alignment of the MNP on their heating properties is due, among others, to the experimental observation that bacteria secreted magnetic nanoparticles—magnetosomes—have an increased heating performance, as compared to their synthetic counterparts, mainly attributable to their chain organization. This observation led several other groups to conduct studies related to the effects of the MNPs controlled assembly at the nanoscale on their hyperthermia properties [52,56,57,58,59]. In a similar attempt, in a recent previous paper, we were able to prove that the pre-alignment of MNPs in a static magnetic field (Supplementary Figure S10), before their immobilization in a solid matrix, lead to an increase up to 40% in the SAR values for manganese and/or zinc ferrites in a ferrimagnetic state (Supplementary Figure S11) [55]. The results obtained for the SP samples described in the present study showed a smaller but clearly measurable increase of the SAR of around 20% for the zinc doped MNPs and almost double, of around 40%, for the MNPs (Figure 7c,d). Although most of the MNPs are in a SP state we consider that the alignment in a static magnetic field, and their organization within chains induces a certain group anisotropy leading to an increase in their heating ability while immobilized in a solid matrix. Our data are in good agreement with very recent published results, demonstrating a ~36% enhancement in heating efficiency in an aqueous magnetic fluid containing tetramethyl-ammonium hydroxide coated SP Fe_3_O_4_ MNPs, after external static bias field (H_DC_ ~ 80 Oe ~ 6.4 kA/m) induced in situ texturing, parallel to the radio frequency alternating magnetic field (RF-AMF) induced in situ texturing [60], the chain formation being confirmed by atomic force microscopy data. A theoretical model demonstrated that such a chain enhances the effective anisotropy energy by 24%. We strongly believe that these results might lead to in situ orientation protocols for further increasing the efficiency of the in vivo magnetic hyperthermia for cancer therapy and eliminating the drawbacks of the highly viscous tissues with low heating efficiency.

One important issue related to our SAR = f(H) curves is the saturation of the SAR values at high AMF amplitudes, this effect being experimentally observed by other research groups on either SP or ferromagnetic MNPs, previously [54,55,61,62,63,64,65,66]. While it is obvious that increasing the AMF amplitude cannot lead to an indefinite increase in the SAR, in the frame of the LRT, the SAR values increase with the square of the AMF amplitude without saturation. However, the LRT is valid only in the low AMF limits, when the Zeeman energy is smaller than the thermal energy (*ξ* < 1). Several approaches were used to explain the saturation while keeping the formalism of LRT. Boskovic et al. showed that SAR saturates with increasing AMF amplitude if one considers the magnetic field dependence of both the Brown and Neel relaxation times [67]. For the Neel relaxation time, Brown proposed in its original paper [68] the following expression for calculating the Neel relaxation time:

(6) $$\frac{1}{\tau_{N}}=\frac{1}{\tau_{0}}\left(1-h^{2}\right)\left\{\left(1+h\right)\exp\left[-\sigma \left(1+h{)}^{2}\right)\right]+\;\left(1-h\right)\exp\left[-\sigma \left(1-h{)}^{2}\right)\right]\right\}$$

where *σ = KV_m_/k_B_T* is a dimensionless parameter with *K* the anisotropy constant and *V_m_* the magnetic volume of the MNPs, and *h = H/H_k_* is the normalized magnetic field, i.e., the AMF amplitude divided by the anisotropy field *H_k_ = M_s_/2K*, and *τ*_0_ is of the order 10^−9^–10^−10^ s. Yoshida and Enpuku [69] proposed the following simple dependence for the Brown relaxation time on the AMF amplitude, derived from the simulations of the Fokker–Plank equation for thermally blocked MNPs in the large AC magnetic fields (ξ < 20):

(7) $$\frac{1}{\tau_{B}}=\frac{1}{\tau_{B0}}\sqrt{1+0.07\xi^{2}}$$

where *τ_B_*_0_ *= 3ηV_h_/k_B_T* with *η* the dynamic viscosity coefficient of the solvent, *V_h_* the hydrodynamic volume of the MNP.

**Figure 8:**
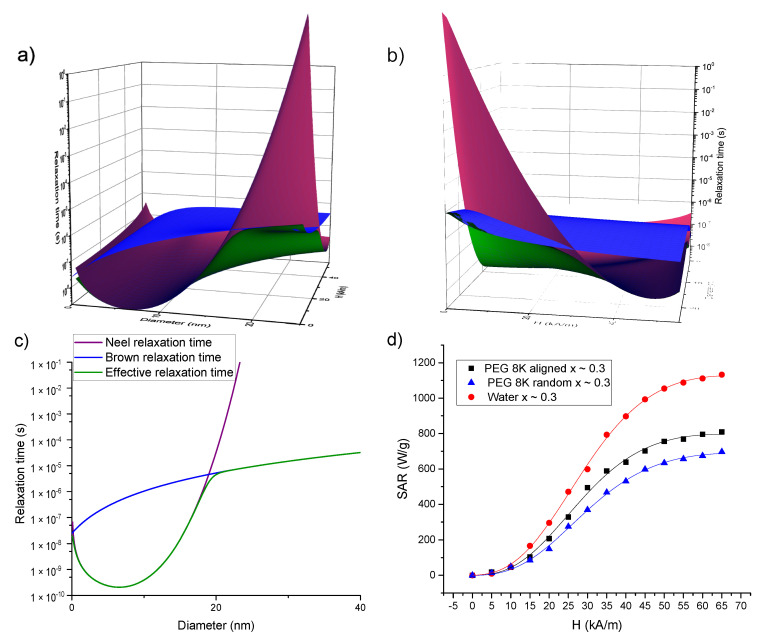
(**a**–**c**) Dependence of the Brown (blue), Neel (purple) and effective (green) relaxation times on the MNPs size and AMF amplitude. In (**a**) the plane showing the time dependence on the MNP size can be compared with the well-known size dependences of the relaxation times in the absence of the field (**c**). In (**b**) one can notice that both Brown and Neel relaxation times decrease by increasing the AFM amplitude, but the dependence on the AFM amplitude is steeper for the Neel relaxation time. (**d**) Fitting the experimental SAR data (dots) of the samples with x ~ 0.3 with a fitting function (lines) given by Equation (12), which takes into account the dependence of both Neel and Brown relaxation times on the AMF amplitude.

Increasing the AMF amplitude leads to a decrease in both relaxation times values, as can be seen in Figure 8a–c where the Brown, Neel and effective relaxation times are plotted for magnetite (M_s_ = 450 kA/m, K = 2 × 10^4^ J/m^3^, and a layer of 2 nm around the MNPs is taken into account to differentiate between the magnetic volume and the hydrodynamic one) using Equations (5)–(7). It is well known that the effective relaxation time is dictated by the shortest one between the Neel and Brown relaxation times. In the absence of an external AMF (or for small ones), the Neel relaxation time is shorter up to 15–20 nm sizes and for larger MNPs, the Brown relaxation prevails. Due to the field dependence of the relaxation times, this landscape is completely changed. As one can observe from Figure 8a–c, for AFM amplitudes above 30 kA/m, the Neel relaxation time (having a steeper dependence on the magnetic field strength) prevails and dictates the effective relaxation even for MNPs with diameters larger than 30–40 nm.

Aiming at explaining the saturation of SAR as a function of the AMF amplitude, we considered a model that takes into account an approximation of the non-linear field dependence of the out of phase magnetization, by using the Langevin function [70]. The fitting function is based on Equations (2) and (3) but the static susceptibility from Equation (4) was replaced by the Langevin function, which takes into account the saturation of the magnetization as a function of the AFM amplitude, leading to the following equation [70]:

(8) $$SAR=\;\Gamma \;\left(coth\xi -\frac{1}{\xi }\right)\frac{\omega \tau }{1+{\left(\omega \tau \right)}^{2}}$$

where the coefficient

(9) $$\Gamma =\;\pi \mu_{0}fH\frac{M_{s}}{\rho }\;.$$

**Figure 9:**
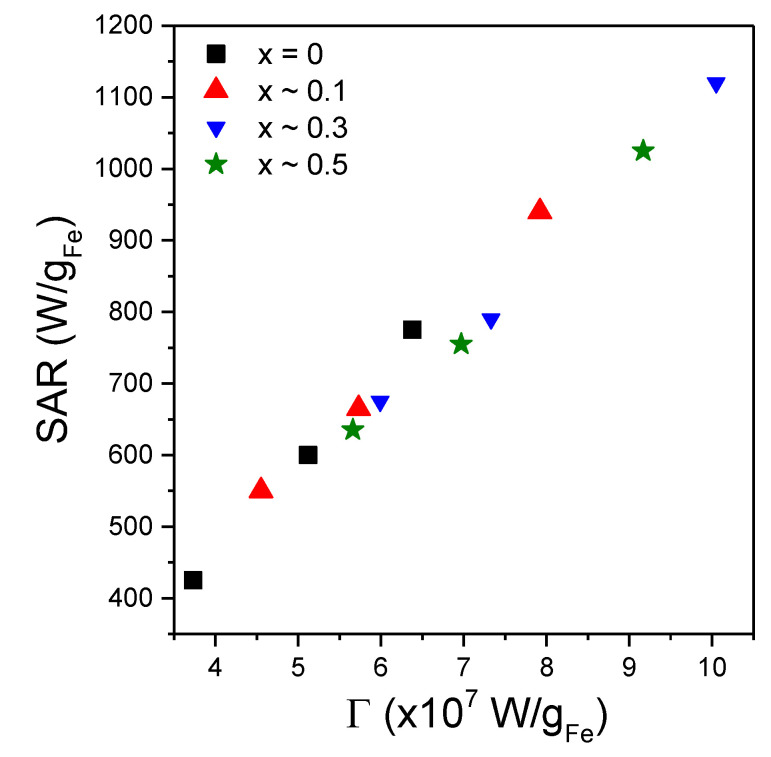
**** Saturation SAR values as a function of the coefficient Γ values resulted from fitting the *SAR* = *f*(*H*) curves by Equation (8), for all four types of MNPs in the three different conditions.

The Brown relaxation time was calculated based on Equation (7). For the Neel relaxation time, it was demonstrated, based on numerical calculations, that its range of validity is restricted to *h* < 0.4 [71]. In our case, for the pure magnetite, the *H_k_* is around 60 kA/m, meaning that in our experimental set-up *h* > 1. As one can easily notice the *τ_N_* presents a singularity point when *h* = 1, which has no physical meaning. Moreover, Equation (6) describes the dependence of *τ_N_* on a static magnetic field while in our case the MNPs are subjected to an AC magnetic field. For these reasons, the experimental data for the Neel relaxation time as a function of AMF amplitude were additionally fitted with an empirical equation resembling Equation (7) (which was derived by Yoshida and Enpuku for the Brownian mechanism) and which was proposed by Dieckhoff et al. [71]:

(10) $$\tau_{N}=\tau_{N0}\sqrt{\left(1+A\xi^{C}\right)}$$

where *τ_N_*_0_ represents the Neel relaxation time in the absence of a magnetic field, and *A* and *C* are fitting parameters. For MNPs of 25 nm in diameter, the AC susceptibility data of Dieckhoff et al. [71] were best fitted with *A* = 1.97 and *C* = 3.18. Therefore, we started our fitting trials with *A* = 2 and *C* = 3. Please note that the ξ parameter depends both on the AMF amplitude and on the MNPs magnetic volume. After many iterations, it appeared that the best fits of the *SAR* = *f*(*H*) curves are obtained if the power of *H* is fixed to 3 allowing the rest of the terms in ξ to have a variable exponent. The values of the M_s_ were set to the values derived from the DC magnetization curves and provided in Table 3. To distinguish the samples measured in water from those in solid PEG 8k, in the latter cases, the dynamic viscosity coefficient was set to 10^100^ Pa∙s. The experimental data and the corresponding fitting curves for the 3 samples with x ~ 0.3 (in water, in solid PEG 8k random and pre-aligned), obtained in the above-described conditions, are presented in Figure 8d while for the other samples (x ~ 0; x ~ 0.1 and x ~ 0.5) in Supplementary Figure S12. The fitting parameters are included in Supplementary Table S1. One can notice a good agreement between the experimental data and the theoretical curves, with coefficients of determination better than 0.999. These results allow us to conclude that saturation in the SAR values could be explained by the changes in the relaxation times with the AFM amplitude. To avoid the over parametrization of the fitting function, the value of the *A* parameter was fixed to 2, while the *C* parameter was allowed to vary. As one can observe from Supplementary Table S1, the obtained values of *C* parameter are in the range 2.81–3.68, most of the values being close to 3, in correlation to those obtained for MNPs of 25 nm in diameter from AC susceptibility data [71]. These results support the hypothesis that the Neel relaxation time depends more steeply on the AMF amplitude (*τ_N_* ~ *H*_max_^−1.5^ from Equation (9) with *C* = 3) as compared with the Brown relaxation time (*τ_B_* ~ *H*_max_^−1^ from Equation (7)). This observation would imply that even for MNPs with larger diameter (D > 20–30 nm), for high AMF amplitudes the magnetization relaxation might be dominated by the Neel mechanism. This result is significant, especially in applications in which the MNPs are in highly viscous environments in which their physical rotation is blocked, like in the case of most biomedical applications (tissues, cell cultures). The diameters of the MNPs resulted from the fittings were in the range 17.66–18.87 nm and are by about 2 nm higher than the mean diameter obtained from TEM and XRD data. For the measurements performed in water, with the MNPs free to rotate, the Brown relaxation contribution to the overall relaxation time is relevant. We noticed that the fitting parameters depend strongly on hydrodynamic diameter, and the best fits were obtained if the thickness of the coating layer at the MNPs surface was taken around 10 nm. There is a very good linear correlation between the SAR max values and the Γ coefficient in Equation (8) as can be seen in Figure 9, for all four samples, in all three conditions. This result supports the idea that Equation (8) can describe correctly the field dependence of SAR in MNPs hyperthermia.

## 3. Materials and Methods

### 3.1. Materials

All the reagents employed in this study were of analytical grade and were used without any further purification. The magnetic nanoparticles were synthesized by using the following products: iron(III) chloride hexahydrate (FeCl_3_∙6H_2_O) (Sigma-Aldrich CAS: 10025-77-1, Darmstadt, Germany), iron(II) chloride tetrahydrate (FeCl_2_∙4H_2_O) (Sigma-Aldrich CAS: 13478-10-9, Darmstadt, Germany), zinc(II) chloride hydrate (ZnCl_2_) (Sigma-Aldrich CAS: 7646-85-7, Darmstadt, Germany) and sodium hydroxide (NaOH) (Sigma-Aldrich CAS: 1310-73-2, Darmstadt, Germany).

### 3.2. Synthesis Method

The conventional co-precipitation synthetic route was applied for the preparation of MNPs, following a similar protocol as described in our previous work [72]. Briefly, 30 mL aqueous solutions of iron and zinc chlorides were prepared separately and stirred for 10 min. The amounts of magnetic precursor used for the synthesis of each type of MNPs are specified in Table 4. The solutions were afterward mixed in a 250 mL three-necked round-bottom flask while stirring at low speed and at T = 50 °C. The precipitation was initiated by adding a certain volume of aqueous solution of NaOH (c = 0.5 M). The reaction temperature and pH during the synthesis were set at 80 °C and 12, respectively. The obtained MNPs were washed several times with double distilled water to remove any soluble salt excess. The powders were afterward lyophilized and stored for further characterization.

### 3.3. Functionalization Method

For ensuring colloidal stability and to avoid the formation of big clusters, MNPs were functionalized using citric acid. Firstly, 20 mg of MNPs powder from each sample was dissolved in a 40 mL aqueous solution of citric acid (c = 0.1 M). Afterward, the solutions were heated at a temperature of 80 °C for 30 min to ensure the grafting of citric acid molecules on the surface of MNPs. Subsequently, the MNPs were magnetically separated and washed with double distilled water and re-dispersed in 10 mL of double-distilled water. Finally, the pH of the solution was adjusted to 7 for hyperthermia measurement.

### 3.4. Characterization Methods

Transmission Electron Microscopy (TEM) was carried out on a JEOL-2100F microscope working at 200 kV. The MNPs were dispersed in a double-distilled water solution and sonicated for 15 min. A 5 μL drop of MNPs aqueous solution was then deposited on a copper grid covered with a holey carbon membrane. After 2 min, the excess water was removed by filter paper and the sample was left to dry under ambient air.

X-ray diffraction (XRD) measurements were carried out on powder samples at room temperature on a Bruker D8 Advance diffractometer (Bruker Co., Ltd, Karlsruhe, Germany) with Cu K_α1 (λ = 0.154056 nm) radiation in the 25–70° (2ϴ) range with a scan step of 0.02°.

Infrared-Spectroscopy (IR) was performed on a Bruker TENSOR II instrument (Bruker Optics Inc., Billerica, MA, USA) in attenuated total reflectance mode, using the platinum attenuated total reflectance (ATR) accessory with a single reflection diamond. The spectra were recorded in the 400–4000 cm^−1^ spectral range, with a resolution of 4 cm^−1^. The MNPs colloids were separated with a magnet and the supernatant was discarded before placing the pellet on the diamond crystal and allowing it to dry. An average spectrum of 16 scans was recorded for each sample.

Dynamic light scattering (DLS) measurements were performed on a Zetasizer Nano ZS90 (Malvern Instruments, Worcestershire, UK) in a 90 configuration. Three cycles of 10 measurements, 5 s each, were performed for each sample at concentrations of 0.2 mg_MNPs_/mL.

Magnetic hysteresis loops were performed on powder samples at 5 K and 300 K and under a range of external fields of ±7 T, using an MPMS3 SQUID magnetometer from Quantum Design Inc.

Hyperthermia measurements were recorded with a magnetic heating system Easy Heat 0224 provided by Ambrell (Scottsville, NY, USA). The samples, usually 0.5 mL of MNPs suspensions in water and solid polyethylene glycol 8000 (PEG 8K), at different concentrations, were placed in a thermally insulated vial, at the centre of an 8-turn coil, connected to the remote heat station of the device. With this setup, AMF with strengths between 5 kA/m and 65 kA/m at a frequency of 355 kHz were generated in the centre of the coil. The temperature was measured using a fibre-optic probe, placed in the centre of the vial, connected to a computer, providing the temperature values each second. The heat performance of MNPs was measured in an environment held at physiological temperature around 37 °C and was quantified by the specific absorption rate (*SAR*), defined as the heat released from a suspension of MNPs in unit time reported to the mass of iron content:

(11) $$SAR=\frac{P}{m_{Fe}}=\frac{Q}{\Delta t\;m_{Fe}}$$

The heat *Q* is calculated as the equivalent heat needed to increase the sample temperature with the same number of *K* in a given time interval:

(12) Q=m c ΔT

where *m* is the mass of the sample subjected to the alternating magnetic field, *c* is the specific heat of the colloid (in our case was approximated with the specific heat of either water or PEG 8k the MNPs contribution to the specific heat being negligible), Δ*T* is the temperature increase of the sample, Δ*t* is the time during which the alternating magnetic field was applied and m_Fe_ is the mass of the iron ions contained in the sample. Due to the non-adiabatic environment, in the SAR determination, it has been taken into account only the linear portion (the first 10 s) of the Δ*T* = *f* (Δ*t*) heating curves. The concentration of MNPs in the colloidal solutions was 1 mg/mL, and since the cations Zn^2+^ do not bear any magnetic moment, values of *m_Fe_* = 0.724, 0.697, 0.644 and 0.592 mg have been considered for samples corresponding to the zinc doping levels x = 0, 0.1, 0.3 and 0.5, respectively. The solvent is water and PEG 8k, thus the *m* = 0.5 g (ρ_water_ = 1 g/mL) or m = 0.5426 g (ρ_PEG8k_ = 1.0852 g/mL) and c_water_ = 4186.8 J/(kg∙K) or c_PEG8k_ = 2135.27 J/(kg∙K).

## 4. Conclusions

By carefully adjusting the amount of magnetic precursors and the pH in the co-precipitation synthesis method, both spherical Fe_3_O_4_ and Zn_x_Fe_3−x_O_4_ MNPs, with different zinc amount (x ~ 0.1, 0.3 and 0.5), have been successfully synthesized. As determined by TEM and XRD data, the four types of MNPs display an average diameter around 16 nm, similar size distribution and crystalline size, thus allowing us to compare their properties and to understand how the Zn doping level affects their structural, magnetic and hyperthermia properties. The EDX data showed that for all the zinc substituted ferrites, the Fe and Zn are homogeneously distributed within the total volume of the MNPs. The MNPs were successfully coated with citric acid as demonstrated by FTIR spectroscopy, increasing their colloidal stability in an aqueous environment. The M_s_ increased with the amount of zinc doping up to x ~ 0.3, while for higher zinc doping (x ~ 0.5), the M_s_ decreased. The increase in M_s_ was explained by the preference of Zn^2+^ ions to occupy the tetrahedral (A) sites, forcing the Fe^3+^ ions to migrate to octahedral (B) sites and replace the divalent Fe^2+^ ions. The mean magnetic diameters of MNPs, determined by fitting the magnetization saturation curves at 300 K, are significantly smaller (around 8–9 nm) as compared to those derived from TEM and XRD data, which implied the existence of the magnetic dead layer around 3 nm surrounding the magnetic core.

The hyperthermia experiments showed that for low AMF amplitudes (0–35 kA/m) the SAR increases with the square of the AMF amplitude as predicted by the LRT theory, whereas above 35 kA/m (40–65 kA/m), a clear saturation effect was observed. The maximum SAR values of MNPs correlated with their M_s_: both the maximum SAR values and M_s_ increase as x is varied between 0 and 0.3 and then decreases for x ~ 0.5. The SAR values decreased for all four samples if the MNPs are embedded in a solid matrix (PEG 8K) due to the blocking of the MNPs physical rotation and of the Brown relaxation mechanism. However, if the MNPs were pre-aligned at high temperature liquid PEG8k, in an external static magnetic field, before the solidification of the suspension, the SAR values increased by 20% in the case of Zn doped ferrites and by 40% in the case of pure magnetite. The performed simulation showed that the saturation of the SAR as a function of the AMF amplitude could be explained by the field dependence of both Brown and Neel relaxation times.

Our results demonstrated that Zn doping improves significantly both the magnetic and the hyperthermia properties of MNPs. Moreover, our results suggested that a further increase in the efficiency of the magnetic hyperthermia might be obtained by chain-like pre-aligned MNPs and that at high amplitude magnetic fields, the Neel relaxation mechanism might be the dominating one, even for MNPs with larger diameters. The latter results might be significant for biomedical applications of MNPs.

## Figures and Tables

**Table 1:** Structural properties of Zn_x_Fe_3−x_O_4_ MNPs.

Zn_x_Fe_3−x_O_4_ MNPs (x)	D_TEM_ (nm)	D_XRD_ (nm)	D_MAG_ and PI * (nm)	a (Å)
0	15.90 ± 0.17	14.30 ± 1.52	8.69 ± 0.03 and 0.16	8.393
0.1	16.15 ± 0.22	15.50 ± 1.89	8.51 ± 0.02 and 0.15	8.402
0.3	16.90 ± 0.21	16.40 ± 1.64	7.76 ± 0.01 and 0.08	8.411
0.5	16.10 ± 0.14	15.30 ± 1.45	8.19 ± 0.03 and 0.07	8.412

* PI = polydispersity index.

**Table 2:** Structural properties of Zn_x_Fe_3−x_O_4_ MNPs.

Theoretical Composition			Experimental Composition by EDX Analysis		
Zn Content	Atomic Percentage (%)		Atomic Percentage (%)		Zn Content
x	Fe	Zn	Fe	Zn	x
0.1	96.67	3.33	97.1 ± 0.5	2.9 ± 0.1	0.09
0.3	90.00	10	91 ± 0.4	9 ± 0.2	0.27
0.5	83.33	16.66	85.1 ± 0.4	14.9 ± 0.2	0.45

**Table 3:** Magnetic properties of Zn_x_Fe_3−x_O_4_ MNPs (0 ≤ x < 0.5).

Sample	5 K					300 K
	M_s_ (Am^2^/kg)	H_c_ (kA/m)	M_r_ (Am^2^/kg)	M_r_/M_s_	K_eff_ (10^4^ × J/m^3^)	M_s_ (Am^2^/kg)
Fe_3_O_4_	76.2	31	19.1	0.25	1.60	65.9
Zn_0.09_Fe_2.91_O_4_	97.1	23	20.5	0.21	1.53	78.5
Zn_0.27_Fe_2.73_O_4_	120.1	19.1	18.4	0.15	1.57	93.4
Zn_0.45_Fe_2.55_O_4_	110.1	21.5	17.3	0.15	1.59	82.1

**Table 4:** Amounts of magnetic precursors used in the synthesis of MNPs.

Zn_x_Fe_3−x_O_4_ MNPs (x)	Magnetic Precursors	Number of Moles (mmol)	Amount (g)
0	FeCl_3_ 6H_2_O FeCl_2_ 4H_2_O	8.80 4.40	2.38 0.88
0.1	FeCl_3_ 6H_2_O FeCl_2_ 4H_2_O ZnCl_2_	8.80 3.96 0.44	2.38 0.79 0.09
0.3	FeCl_3_ 6H_2_O FeCl_2_ 4H_2_O ZnCl_2_	8.80 3.08 1.32	2.38 0.61 0.18
0.5	FeCl_3_ 6H_2_O FeCl_2_ 4H_2_O ZnCl_2_	8.80 2.20 2.20	2.38 0.88 0.29

## Supplementary Materials

Supplementary materials can be found at https://www.mdpi.com/1422-0067/21/20/7775/s1.

## Abbreviations

MNPs	Magnetic nanoparticles
T_B_	Blocking temperature
SP	Superparamagnetic
SPIONs	Superparamagnetic iron oxide nanoparticles
MRI	Magnetic Resonance Imaging
MH	Magnetic Hyperthermia
M_s_	Saturation magnetization
FCC	Face Centred Cubic
AMF	Alternating Magnetic Field
SAR	Specific Absorption Rate
TEM	Transmission Electron Microscopy
XRD	X-ray Diffraction
EDX	Energy dispersive X-ray
FT-IR	Fourier Transform Infrared
H_c_	Coercive field
M_r_	Magnetic remanence
ZFC	Zero Field Cooled
FC	Field Cooled
LRT	Linear Response Theory
AC	Alternating Current
