# Supplementary material for: Quantitative Analysis of the Specific Absorption Rate Dependence on the Magnetic Field Strength in ZnxFe3−xO4 Nanoparticles
Source: Int J Mol Sci. 2020 Oct 21;21(20):7775. doi: 10.3390/ijms21207775 (PMC7590026; doi:10.3390/ijms21207775)
Supplement: Supplementary file 1 [file ijms-21-07775-s001.pdf]

Supplementary Materials

Quantitative Analysis of the Specific Absorption Rate Dependence on the Magnetic Field Strength in $Zn_xFe_{3-x}O_4$ Nanoparticles

Mohamed Alae Ait Kerroum [1,2,†], Cristian Iacovita [3,†], Walid Baaziz [1], Dris Ihiawakrim [1], Guillaume Rogez [1], Mohammed Benaissa [2], Constantin Mihai Lucaciu [3,*] and Ovidiu Ersen [1,*]

[1] Institut de Physique et Chimie des Matériaux de Strasbourg (IPCMS), UMR 7504 CNRS-Université de Strasbourg, 23 rue du Loess BP 43, 67034 Strasbourg Cedex 2, France.; mohamed-alae.ait-kerroum@ipcms.unistra.fr (M.A.A.K); walid.baaziz@ipcms.unistra.fr (W.B.); dris.ihiawakrim@ipcms.unistra.fr (D.I.); guillaume.rogez@ipcms.unistra.fr (G.R.)

[2] Laboratoire de Matière Condensée et Sciences Interdisciplinaires (LaMCScI), Faculty of Sciences, BP 1014 RP, Mohammed V University in Rabat, 10000 Rabat, Morocco; benaissa@fsr.ac.ma

[3] Department of Pharmaceutical Physics-Biophysics, Faculty of Pharmacy, "Iuliu Hatieganu" University of Medicine and Pharmacy, Pasteur 6, 400349 Cluj-Napoca, Romania.; cristian.iacovita@umfcluj.ro (C.I.)

* Correspondence: clucaciu@umfcluj.ro (C.M.L); ovidiu.ersen@ipcms.unistra.fr (O.E.); Tel.: +00-40-744-647-854 (C.M.L.); +00-33-03-88-10-70-28 (O.E.)

† These authors contributed equally to this work.

Outline:

S1. Shift of diffraction peaks towards lower angles with increased zinc content

S2. EDX mapped images

S3. FT-IR spectra of bare MNPs

S4. Dynamic Light Scattering measurements

S5. Field Cooled and Zero Field Cooled curves for the four types of MNPs

S6. Magnetization saturation curves fitting using a log-normal distribution and the Langevin function

S7. Heating curves [T = f(t) curves] for the four types of MNPs dispersed in water

S8. Heating curves [T = f(t) cuves] for the four types of MNPs randomly dispersed in PEG8K

S9. Heating curves [T = f(t) curves] for the four types of MNPs pre-aligned in a static magnetic field of 15 mT while being dispersed in PEG8K

S10. Alignment of the MNPs in a static magnetic fields

S11. SAR values dependence on the magnetization saturation

S12. Fitting of the SAR = f(H) curves with equation 12 form the main text

S13. Saturation SAR values as a function of the coefficient Γ value

Table S1. The parameters derived from the fitting of SAR = f(H) curves

S1. Shift of diffraction peaks towards lower angles with increased zinc content

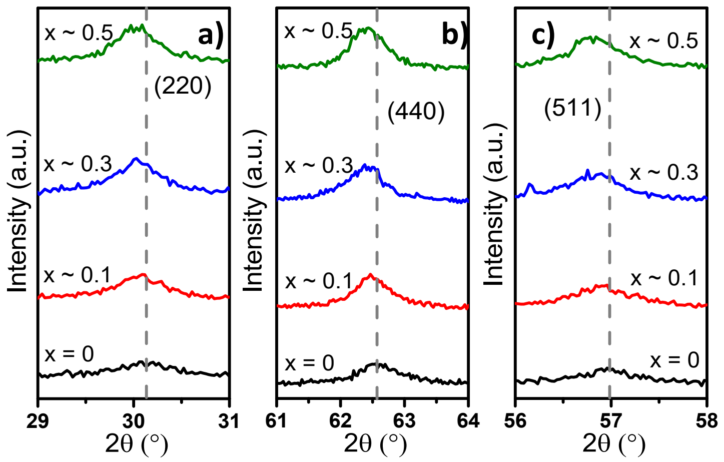

Figure S1. Zooms on the (220) (**a**), (511) (**b**) and (440) (**c**) diffraction peak regions of XRD diffraction patterns of $Zn_xFe_{3-x}O_4$ MNPs with different zinc doping level ($0 \leq x < 0.5$).

S2. EDX mapped images

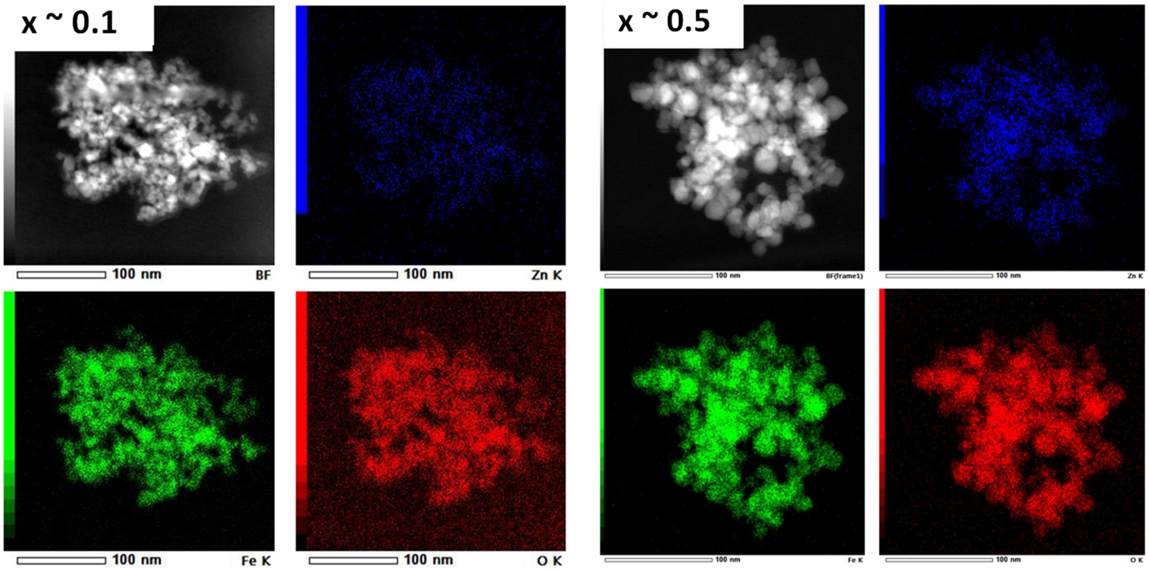

(**a**) (**b**)

Figure S2. TEM image of $Zn_xFe_{3-x}O_4$ MNPs with x ~ 0.1 (**a**) and x ~ 0.5 (**b**) and the corresponding EDX mapped images for Zinc (blue), Iron (green) and Oxygen (red).

S3. FT-IR spectra of bare MNPs

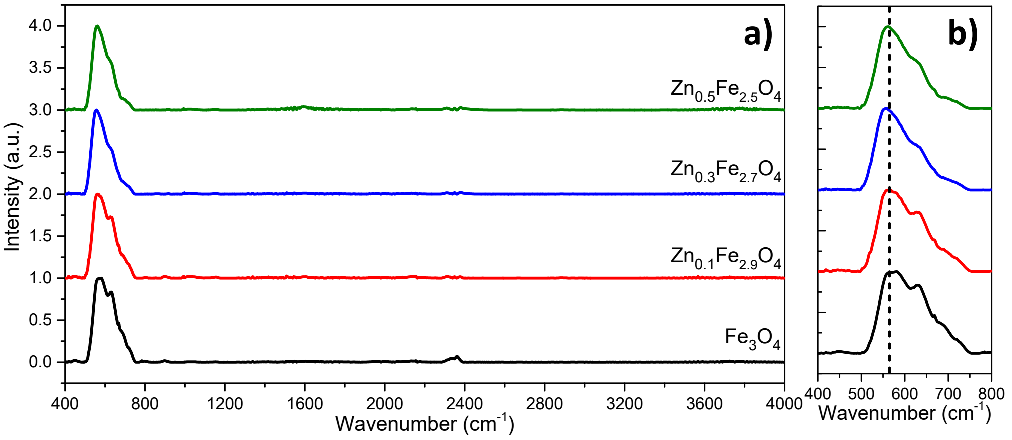

Figure S3. (**a**) FT-IR spectra of uncoated $Zn_xFe_{3-x}O_4$ MNPs ($0 \le x < 0.5$) and (**b**) Zoom in the 400–800 cm^{-1} region of FT-IR spectra. The spectra are normalized to the highest absorption band and shifted for clarity.

S4. Dynamic Light Scattering measurements

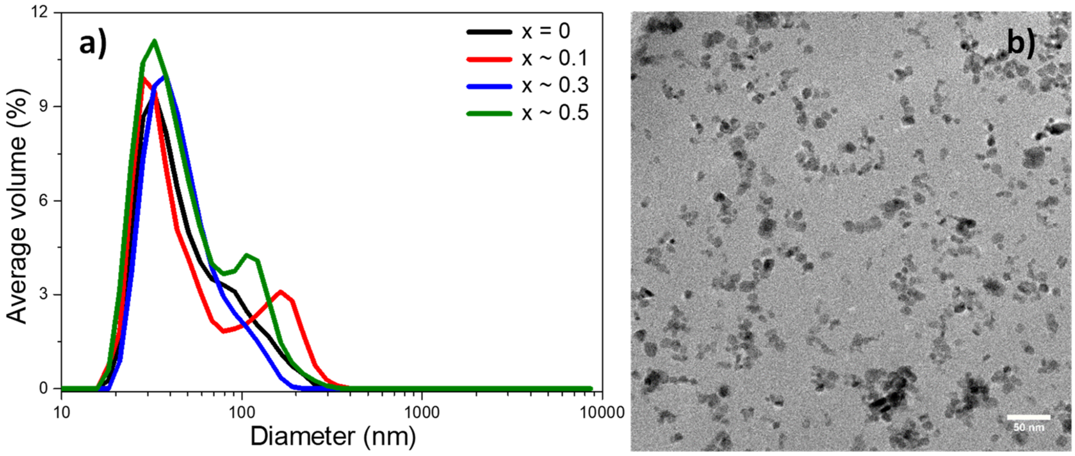

Figure S4. (**a**) FT-IR spectra of uncoated $Zn_xFe_{3-x}O_4$ MNPs ($0 \le x < 0.5$) and (**b**) Zoom in the 400–800 cm^{-1} region of FT-IR spectra. The spectra are normalized to the highest absorption band and shifted for clarity.

S5. Field Cooled and Zero Field Cooled curves for the four types of MNPs

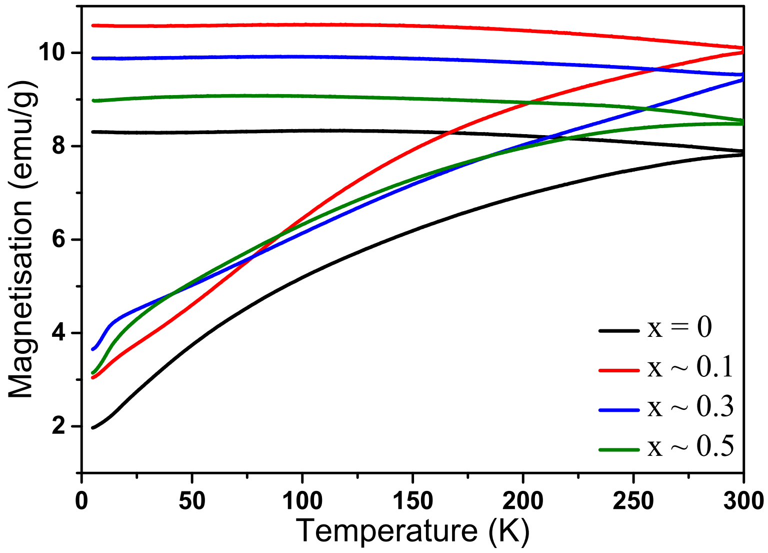

Figure S5. ZFC and FC magnetization curves of $Zn_xFe_{3-x}O_4$ MNPs ($0 \leq x < 5$).

S6. Magnetization saturation curves fitting using a log-normal distribution and the Langevin function

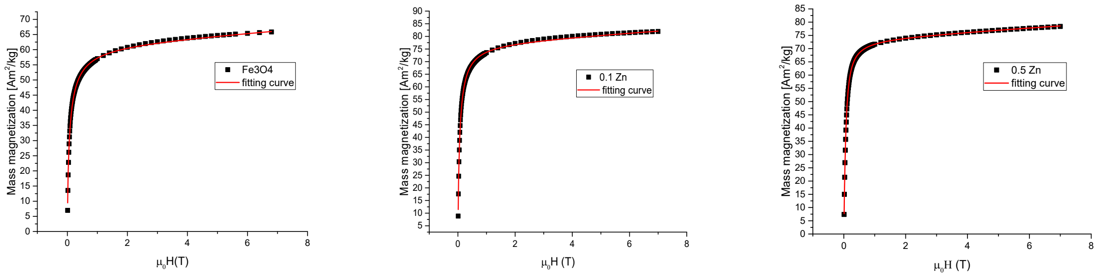

(**a**) (**b**) (**c**)

Figure S6. Magnetization curve fitting for the $Zn_xFe_{3-x}O_4$ MNPs with x = 0 (**a**), x ~ 0.1 (**b**) and x ~ 0.5 (**c**) at 300 K, the black squares represent the experimental data and the red lines are the fitting curves.

S7. Heating curves [T = f(t) curves] for the four types of MNPs dispersed in water

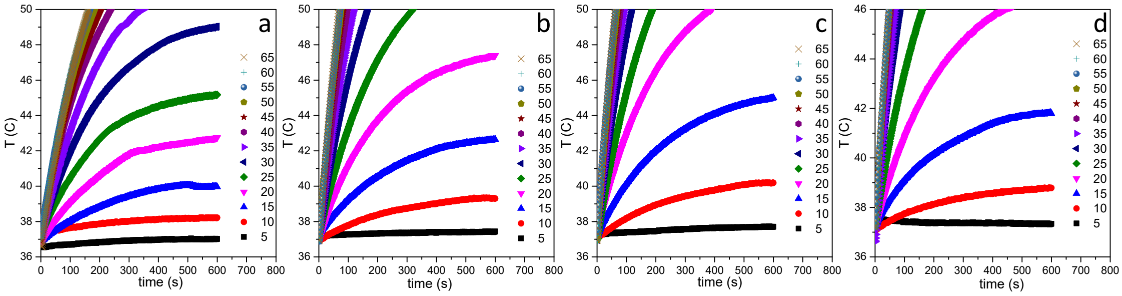

Figure S7. Heating curves of $Zn_xFe_{3-x}O_4$ MNPs with (**a**) x = 0, (**b**) x ~ 0.1, (**c**) x ~ 0.3 and (**d**) x ~ 0.5 dispersed in water at a concentration of 1 mg/mL, recorded as a function of AC magnetic field amplitudes at 355 kHz.

S8. SAR values dependence on the magnetization saturation

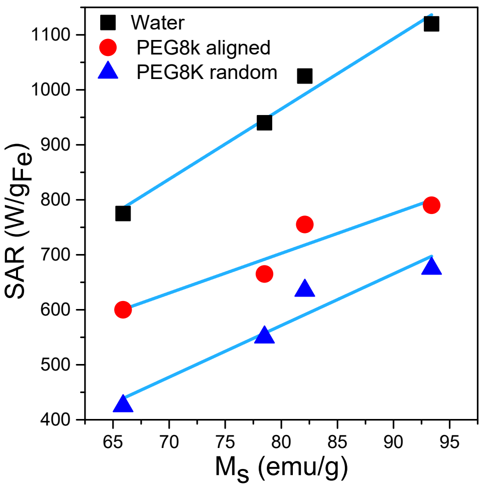

Figure S8. The saturation SAR values of four types of MNPs in both media as a function of the magnetization saturation of MNPs. The blue lines represent linear fits.

S9. Heating curves [T = f(t) cuves] for the four types of MNPs randomly dispersed in PEG 8K

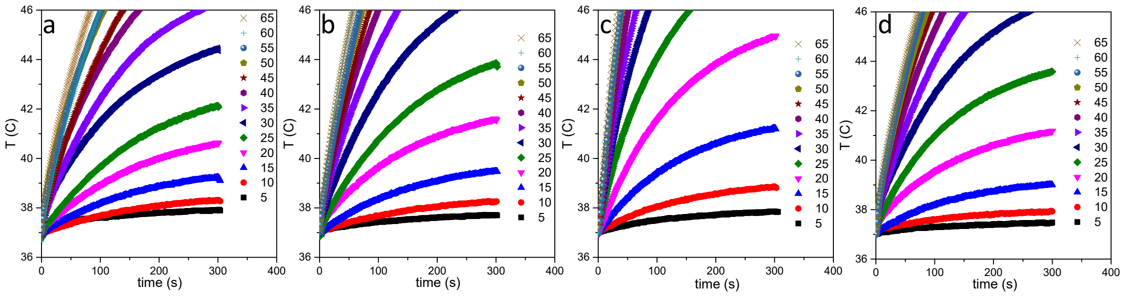

Figure S9. Heating curves of $Zn_xFe_{3-x}O_4$ MNPs with (**a**) x = 0, (**b**) x ~ 0.1, (**c**) x ~ 0.3 and (**d**) x ~ 0.5 randomly dispersed in PEG 8k at a concentration of 1 mg/mL, recorded as a function of AC magnetic field amplitudes at 355 kHz.

S10. Alignment of the MNPs in a static magnetic fields

The MNPs at a concentration of 1 mg/mL dispersed in water were collected at the bottom of the vial by a magnet; the water was discharged and 0.5 mL liquid PEG 8K heated at 80 °C was introduced. The samples were immediately sonicated for 10 minutes in an ultra-sonication bath heated at 80 °C. Right after the samples were placed in the middle of the distance between two 1 cm cubic Neodymium magnets separated by 7 cm. The magnetic induction measured with a Gaussmeter is almost constant in a region of around 1 cm in the center of the system (between 3 cm and 4 cm from one magnet) according to the calibration curve provided in Supplementary Figure S6. The samples were left to solidify under a 15 mT static magnetic field.

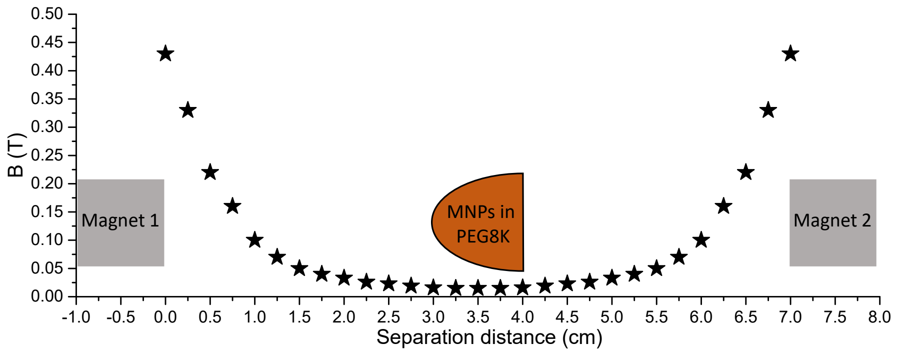

Figure S10. Magnetic induction calibration curve between two neodymium (Ne-Fe-B) magnets separated by 7 cm one from the other.

S11. Heating curves [T = f(t) curves] for the four types of MNPs pre-aligned in a static magnetic field of 15 mT while being dispersed in PEG 8K

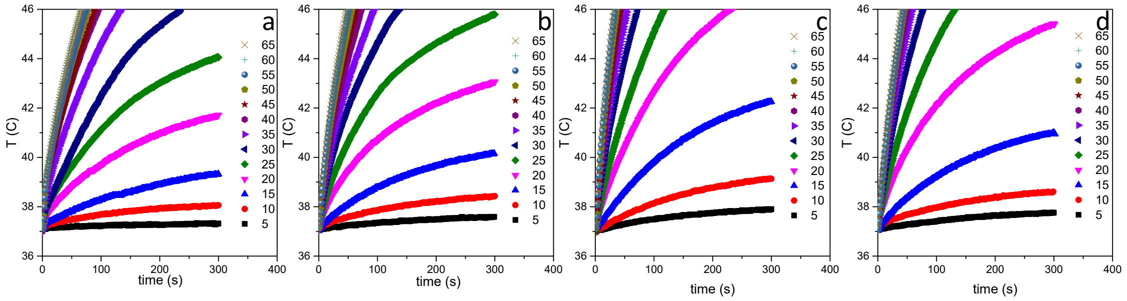

Figure S11. Heating curves of $Zn_xFe_{3-x}O_4$ MNPs with (**a**) x = 0, (**b**) x ~ 0.1, (**c**) x ~ 0.3 and (**d**) x ~ 0.5 pre-aligned in a static magnetic field of 15 mT while being dispersed in PEG 8k at a concentration of 1 mg/mL, recorded as a function of AC magnetic field amplitudes at 355 kHz.

S12. Fitting of the SAR = f(H) curves with equation 12 from the main text

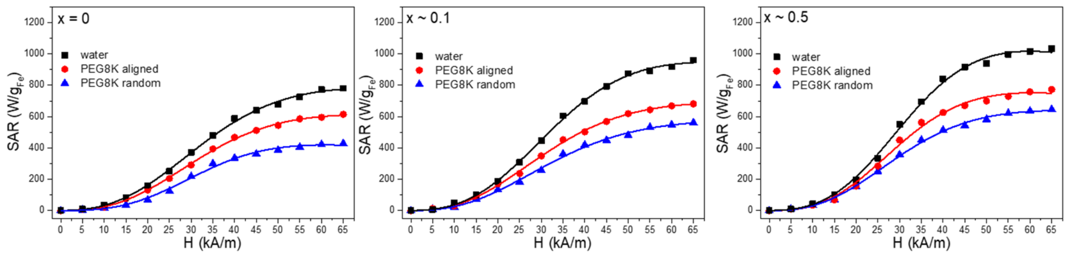

(**a**) (**b**) (**c**)

Figure S12. Fitting the experimental SAR data (dots) of the samples with x = 0 (**a**), x ~ 0.1 (**b**) and x ~ 0.5 (**c**) with a fitting function (lines) given by equation 12 from the main text, which takes into account the dependence of both Neel and Brown relaxation times on the AMF amplitude.

Table S1. The parameters derived from the fitting of SAR = f(H) curves.

$Zn_xFe_{3-x}O_4$ MNPs (x)	Condition	Γ (× 10^7 W/g_{Fe})	C	D (nm)	SAR_{MAX} (W/g_{Fe})
x = 0	Water	6.38 ± 0.20	3.17	18.76 ± 0.76	780
	PEG8K aligned	5.12 ± 0.12	3.55	17.66 ± 0.06	620
	PEG8K random	3.73 ± 0.15	3.07	18.87 ± 0.08	420
X ~ 0.1	Water	7.92 ± 0.19	3.37	17.16 ± 0.07	1000
	PEG8K aligned	5.73 ± 0.14	2.87	17.39 ± 0.06	680
	PEG8K random	4.55 ± 0.12	2.81	17.37 ± 0.11	520
x ~ 0.3	Water	10.05 ± 0.21	3.07	17.85 ± 0.05	1150
	PEG8K aligned	7.33 ± 0.23	2.98	17.66 ± 0.07	800
	PEG8K random	5.99 ± 0.15	2.97	17.67 ± 0.06	660
x ~ 0.5	Water	9.17 ± 0.3	3.68	18.72 ± 0.19	1050
	PEG8K aligned	6.97 ± 0.27	3.31	18.10 ± 0.07	750
	PEG8K random	5.66 ± 0.11	3.02	17.66 ± 0.06	600
